# Enhanced HMGB1 Expression May Contribute to Th17 Cells Activation in Rheumatoid Arthritis

**DOI:** 10.1155/2012/295081

**Published:** 2011-10-26

**Authors:** Yan Shi, Siamak Sandoghchian Shotorbani, Zhaoliang Su, Yanfang Liu, Jia Tong, Dong Zheng, Jianguo Chen, Yingzhao Liu, Yan Xu, Zhijun Jiao, Shengjun Wang, Liwei Lu, Xinxiang Huang, Huaxi Xu

**Affiliations:** ^1^Department of Immunology, Institute of Laboratory Medicine, Jiangsu University, Xuefu Road 301, Zhenjiang 212013, China; ^2^Suzhou Municipal Hospital, Suzhou 215002, China; ^3^The Affiliated People's Hospital of Jiangsu University, Zhenjiang 212002, China

## Abstract

Rheumatoid arthritis(RA) is a common autoimmune disease associated with Th17 cells, but what about the effect of high-mobility group box chromosomal protein 1 (HMGB1) and the relationship between Th17-associated factors and HMGB1 in RA remains unknown. In the present study, we investigated the mRNA levels of HMGB1, ROR*γ*t, and IL-17 in peripheral blood mononuclear cells (PBMCs) from patients with rheumatoid arthritis by quantitative real-time PCR (RT-qPCR), and the concentrations of HMGB1, IL-17, and IL-23 in plasma were detected by ELISA. And then, the effect of HMGB1 on Th17 cells differentiation was analyzed *in vitro.* Our clinical studies showed that the mRNAs of HMGB1, ROR*γ*t, and IL-17 in patients were higher than that in health control (*P* < 0.05), especially in active RA patients (*P* < 0.05). The plasma HMGB1, IL-17, and IL-23 in RA patients were also higher than that in health control (*P* < 0.05); there was a positive correlation between the expression levels of HMGB1 and the amount of CRP, ERS, and RF in plasma. *In vitro*, the IL-17-produced CD4^+^T cells were increased with 100 ng/mL rHMGB1 for 12h, which indicated that the increased HMGB1 might contribute to Th17 cells activation in RA patients.

## 1. Introduction

Rheumatoid arthritis (RA) is an autoimmune disease characterized by chronic inflammation in the small joints leading to the destruction of articular cartilage and bone. TNF-*α*, IL-1, and IL-17 as well as T, B lymphocytes and macrophages are implicated in the pathogenesis of RA [1–3]. Recently, high-mobility group box chromosomal protein 1 (HMGB1), a nonhistone nuclear DNA-binding protein, is proved to be a potent proinflammatory mediator in rheumatoid arthritis [4–7]. Increased HMGB1 was found in the joints of RA patients [8–10], and the HMGB1 transferred into health mouse joint could induce the arthritis [11]. HMGB1 is secreted or released from lymphocytes, dead and/or apoptosis cells [12–15]. Previous studies have showed that HMGB1 in milieu could contribute proinflammatory such as IL-6, IL-1*β*, and IL-10 secretion by macrophages, sustain inflammation [15]. It is clear that IL-6 and IL-1*β* can prime the naïve CD4^+^T cells differentiation into Th17 cells [16].

Whether HMGB1 involved in the pathogenesis of RA by promoting the Th17 cells activation was unclear. In the present study, we examined the expression levels of HMGB1 and Th17-associated factors in RA patients, analyzed the relationship between them, and explored the potentiality of HMGB1 in Th17 differentiation *in vitro.*

## 2. Patients and Methods

### 2.1. Patients

80 patients with RA enrolled in the affiliated hospital of Jangsu University were included in this study from January 2008 to September 2009. Among 80 patients, 59 females and 21 males, ranged from 36 to 80 years old. 48 patients were in active phase, and 32 patients were in inactive phase. Diagnoses were established according to the American College of Rheumatology (ACR) criteria [17] and the disease activity score calculated for 28 joints (DAS28). 48 patients in active phase untreament during the past 2 years which did not accompany other chronic diseases; all the 48 patients included 8 males and 40 females, the age was 43 ± 10 years, the disease duration was 10.3 ± 4.5 months, and the range of DAS28 was 4.21–6.32 (the mean was 5.60 ± 0.78); the RA patients in inactive phase were 32 cases, 6 males and 26 females, the age was 49 ± 13 years, the course of disease was 40.1 ± 25.7 months, and the DAS28 range was 1.92 to 0.67 (the average was 1.75 ± 0.23). 50 healthy volunteers, 38 females and 12 males ranged from 28 to 41 years old, acted as control. This study was approved by the ethical committee of the Affiliated Hospital of Jiangsu University. All individuals were informed consensus.

### 2.2. Reagent

rHMGB1 was expressed in *Escherichia coli *(*E.coli*) and purified by Ni-column. The control protein eGFP was produced from *E.coli* and purified by the same method.

### 2.3. Blood Samples

Peripheral blood samples were collected from healthy volunteers and patients. The collection tubes contained 0.2 mL sodium heparin. The blood samples were centrifugalized at 1000 r/min 4°C for 5 min, then the supernatant was collected and stored at −70°C for use, and sediment were separated from PBMCs by standard Ficoll-Hypaque density centrifugation. TRIzol was added to the PBMCs for total RNA.

### 2.4. Primers Design

According to Genbank sequences, the primers were designed by Premier 5.0 software and synthesized by Shanghai Sangon Biological Engineering Technology and Service Company. All sequences of primers were shown in Table 1.

### 2.5. RNA Extraction and cDNA Synthesis

Following the manufacturer's instructions, total RNA from PBMCs was extracted with Trizol (Invitrogen, USA). cDNA was synthesised with reverse transcription reagent kits (TOYOBO, Japan). All RNA samples were heated at 65°C for 10 min to denature the secondary structure with the template then put in ice for 5 min. Total RNA (1 *μ*g) was reversely transcribed in a total volume of 20 *μ*L, containing Oligo (dT) 1 *μ*L, dNTP (10 mM) 2 *μ*L, 5 × RT buffer 4 *μ*L, ReverTraAce (100 U/*μ*L) 1 *μ*L, RNase Inhibitor 1 *μ*L, DEPC free H_2_O add up to 20 *μ*L, response conditions: 42°C for 20 min; 99°C for 5 min; 4°C for 5 min. The cDNA was stored at −20°C.

### 2.6. Construction of Recombinant Plasmid Calibrator

PCR amplification was performed in the Thermon Hybaid System (Eppendorf, USA). The program consisted of an initial denaturation step for 5 min at 94°C followed by 30 cycles, with each cycle consisting of a 30 s denaturing step at 94°C, a 30 s annealing at 56°C and a 30 s extension at 72°C. The reaction was completed by a final 5 min extension at 72°C. Purified HMGB1, ROR*γ*t, IL-17, and *β*-actin PCR fragments were transformed to PMD18-T vector (Invitrogen, USA) to establish recombinant plasmids PMD18-HMGB1, ROR*γ*t, IL-17, and *β*-actin. All these recombinant plasmids were transformed into competent *E. coli* DH5*α*, transferred on a 1.5% agar Amp-resistant plate, and then cultured at 37°C for 12 ~ 14 h. Positive clones were initially identified by sequencing. Part of positive clones were further amplified and extracted and accurately quantified with a nucleic acid-protein ultraviolet instrument. 10-fold serial dilution of the recombinant plasmid DNAs were used as calibrator and stored at −20°C until use.

### 2.7. RT-qPCR-Detected Objective Genes Expression

The objective genes expression (HMGB1, ROR*γ*t, and IL-17) were detected by quantitative real-time polymerase chain reaction (RT-qPCR), and all samples were calibrated by *β*-actin. All PCR reactions were performed using the Rotor-Gene 6000 System (Corbett Research, Australia) in a total volume of 20 *μ*L, containing 1 *μ*L cDNA, 10 *μ*L 2 × sybr1 premix (Takara, China), 0.3 *μ*L 10 *μ*M each primer, and 8.4 *μ*L water. The specificity of the amplification products was controlled using a melting curve analysis. The copy number of ROR*γ*t, IL-17, and *β*-actin transcripts in samples was calculated with the Corbett software according to corresponding standard curves. The copy number of gene/%**β**-actin represented the ratio of the gene. A no-template negative control was also included in each experiment, and all samples were measured in triplicate.

### 2.8. The Function of rHMGB1 in Th17 Differentiation In Vitro

CD4^+^T cells from C57BL/6 mice spleen were prepared by magnetic column; 1 × 10^6^/well cells were put into precoating 24-well plates by anti-CD3, anti-CD28, and cultured in PRIM-1640 including 10% FCS at 5% CO_2_, 37°C, the cells were stimulated with different dose of rHMGB1. The eGFP and LPS from *E. coli* was used as controls. Collected cells after 0, 3, 6, 9, 12, 24, and 48 hours and supernatants were used to detect the related cytokines as previously described. 

### 2.9. Enzyme-Linked Immunosorbent Assays (ELISAs) for IL-17 and IL-23

The levels of HMGB1, IL-17, and IL-23 in plasma or cell culture supernatants were measured by ELISAs, following the manufacturer's protocols (eBioscience, USA). All samples were measured in triplicate.

### 2.10. Flow Cytometry Analysis

The procedures of flow cytometry analysis were performed as described elsewhere [18]. Briefly, 1 × 10^6^ PBMCs were stained with anti-CD3-PE-cy5 (eBioscience), anti-CD8-FITC (eBioscience), and anti-IL-17-PE (eBioscience). 1 × 10^6^ CD4^+^T cells from the spleen of mice were stained with anti-IL-17-FITC (eBioscience). The stained cells were applied for data acquisition on Coulter EPICS XL Cytometer (Beckman Coulter) and analyzed by software WinMDI (version 2.9).

### 2.11. Statistical Analysis

All statistical analysis were performed using SPSS17.0 statistical analysis software. Data are expressed as the mean ± standard deviation (SD) in text and figures. Comparisons between paired or unpaired groups were performed using the appropriate Student's *t*-test. For nonparametric data, differences between two groups were analyzed by the Mann-Whitney test. Spearman's correlation was used to test correlation between two continuous variables. *P  *<  0.05 was considered to be statistically significant.

## 3. Results

### 3.1. Electrophoresis Identification the Amplicons

The amplicon length of HMGB1, ROR*γ*t, IL-17, and *β*-actin was 233, 171, 231, and 265 bp, respectively, and it was consistent with the expected data. The positive clone recombinant plasmid was identified by sequencing. These objective gene sequences were in accordance with Genbank seqence (detailed data not shown).

### 3.2. The Linear Range and Reproducibility

The detection range of recombinant plasmid DNAs was from 10 to 10^8^ copies, and the coefficients of variation values ranged from 2.20% to 8.32%. Amplification efficiency ranged from 0.88 to 0.92, and *r* ^2^  >  0.99.

### 3.3. Levels of HMGB1, ROR*γ*t, and IL-17 mRNA in RA Patients

**Figure 1:**
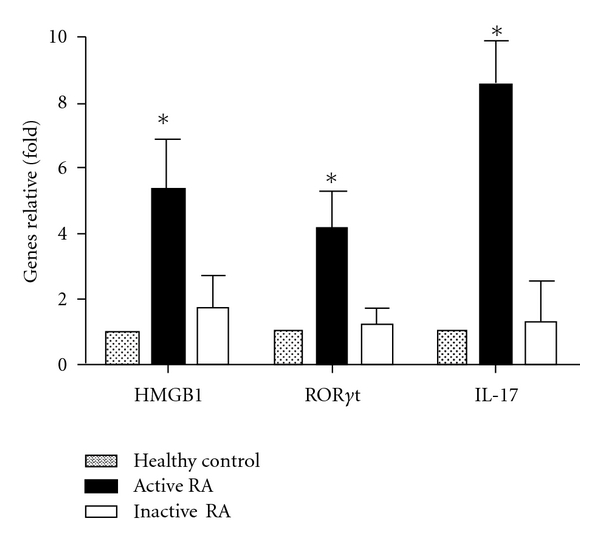
Expression of gene ratio by RT-qPCR. The mRNA expression level were determined by RT-qPCR, the values were expressed as the fold of the healthy control. the ratio of target genes used the healthy control as 1. *P < 0.05 compared with the healthy control and inactive RA group.

The expression levels of HMGB1, ROR*γ*t, and IL-17 mRNA from RA patients and healthy controls were measured by RT-qPCR. As shown in Figure 1, the mRNAs of Th17-associated cytokines and transcription factor were significantly increased in PBMCs from RA patients, especially in active phase of RA patients, and it was quite different from that in inactive phase of patients and healthy controls (*P* < 0.05).

### 3.4. The Correlations between the mRNA Levels of HMGB1 and Th-17 Cells-Related Factors

**Figure 2:**
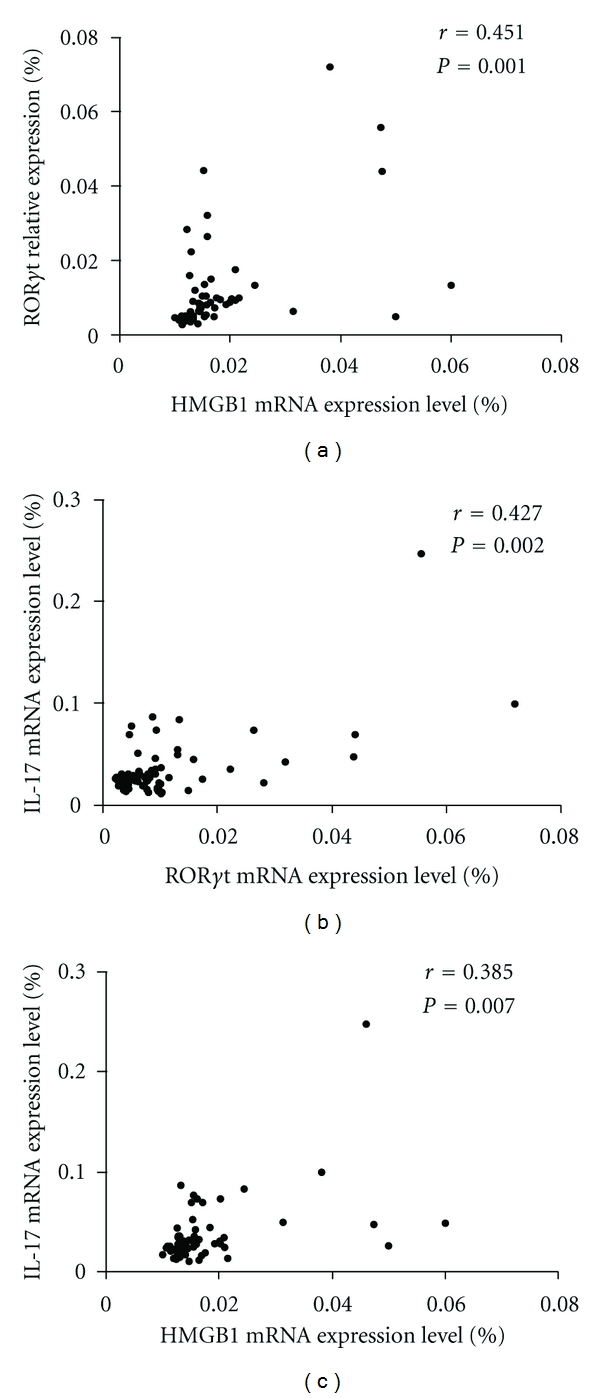
Correlations of IL-17 and ROR*γ*t and HMGB 1 mRNA level in RA active patients. The mRNA expression levels as determined by RT-qPCR, the values were expressed as the target genes versus *β*-actin mRNA expression.

To assess the relationships between the mRNA levels of HMGB1 and Th-17 cells-related factors in RA patients. We examined the correlation between the mRNA levels of HMGB1 and Th17 cells-related factors in PBMCs of RA patients. There was a significantly positive correlation among them (Figure 2).

### 3.5. Increased Cytokine Concentrations in Plasma from Patients with RA

Concentrations of plasma HMGB1, IL-23, and IL-17 measured by ELISA in each group are shown in Table 2. The Th17 cell-associated cytokines were significantly increased in plasma from active phase of RA patients, but no obvious difference between inactive RA and healthy controls. The correlations analysis among HMGB1, IL-23, IL-17, and other clinical targets in the serum of active RA patients showed that there was a significantly positive correlation (Table 3).

### 3.6. Increased Frequencies of CD3^+^CD8^−^IL-17^+^T Cells in PBMCs from RA Patients

**Figure 3:**
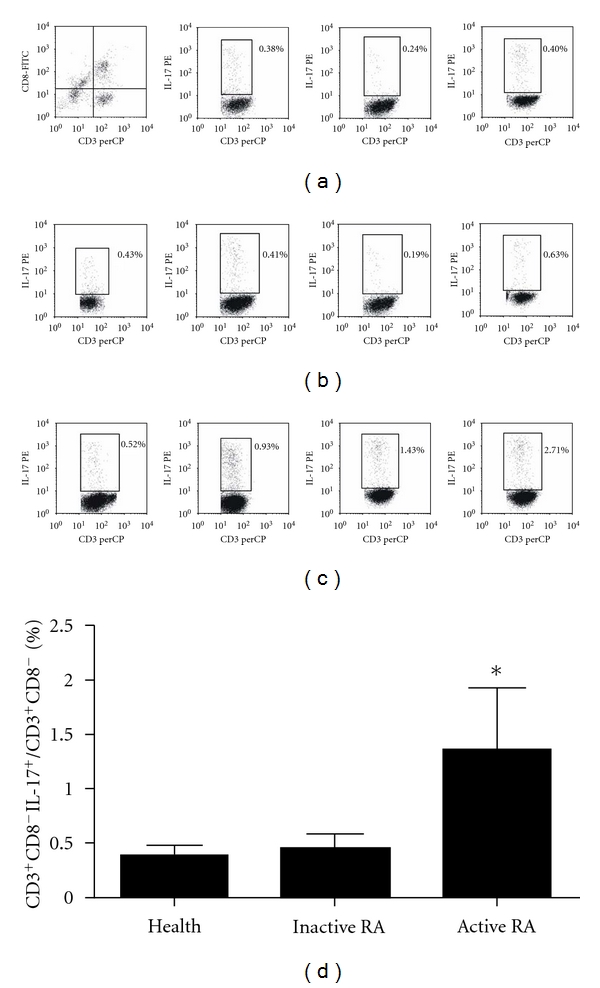
FACS analyzed the CD3^+^CD8^−^IL-17^+^ cell ratio in RA patient The PBMCs were isolated by standard Ficoll-Hypaque density centrifugation. The cells were stained by anti-CD3-PE-cy5, anti-CD8-FITC, and anti-IL-17-PE. (a) First figure presented CD3^+^CD8^−^T cells were considered CD4^+^T cells in region RL, and the other three were presented healthy controls. (b) Representative IL-17 expression in CD3^+^CD8^−^T subsets from RA patients in inactive phase. (c) Representative IL-17 expression in CD3^+^CD8^−^T subsets from RA patients in active phase. (d) The results were shown as means ± SD. *P < 0.05 compared with the control group.

Flow cytometry was used to assess frequencies of CD3^+^CD8^−^IL17^+^T cells in PBMCs from patients and controls, and the results showed that CD3^+^CD8^−^IL17^+^T cells in active phase of patients (1.36 ± 0.98%) were higher than those in controls (0.39 ± 0.16%), and the difference was statistical significance (*P  *<  0.05), whereas there was no significant difference was found between inactive phase of patients (0.45 ± 0.23%) and controls (Figure 3).

### 3.7. The mRNA Expression Levels of Th17 Cell-Related Factors in rHMGB1-Stimulated Mice CD4^+^T In Vitro

**Figure 4:**
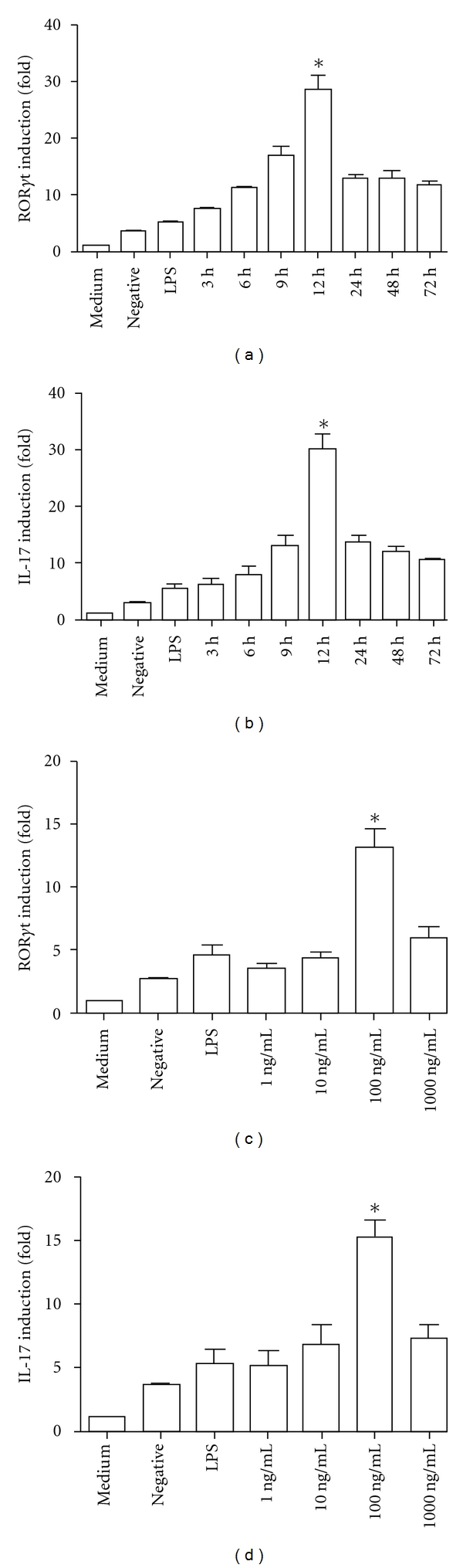
Th17 cell-related factors expression stimulated by HMGB 1 *in vitro*. Th17 cell-related factors expression by HMGB 1 stimulus *in vitro* were detected by RT-qPCR. The CD4^+^T cells were isolated from mouse spleen, preactivated by anti-CD3 and anti-CD28, and then added to rHMGB 1. (a) ROR*γ*t mRNA expression levels after rHMGB1 stimulating in different time; (b) IL-17 mRNA expression leves after rHMGB 1 stimulus; (c) ROR*γ*t mRNA expression levels after rHMGB 1 stimulus; (d) IL-17 mRNA expression levels after CD4^+^T cells stimulated by rHMGB 1 stimulus. RT-qPCR analysis for target genes versus *β*-actin mRNA expression, the ratios of target genes used the control as 1. Data from 3 independent experiments were presented as means ± s.d. *P < 0.05 versus control.

To further confirm the relationship between HMGB1 and Th17 cells, rHMGB1 was used to stimulate CD4^+^T cells *in vitro* and then to detect the mRNA levels of Th17 cell-associated factors by RT-qPCR. Our data indicated that rHMGB1 could enhance the expression levels of Th17 cell-related factors, and the levels changed with the dose and time stimulated by rHMGB1. In the 0.1–100 ng/mL rHMGB1 stimulus dose range, Th17-related factors expression was a dose dependence, 100 ng/mL was the best concentration. Cells were collected at the different points after rHMGB1 stimulation, and Th17 cell-related factors was up to the peak at 12 h (Figure 4).

### 3.8. CD4^+^IL-17^+^T Cells Ratio Was Increased under the rHMGB1 Stimulation

**Figure 5:**
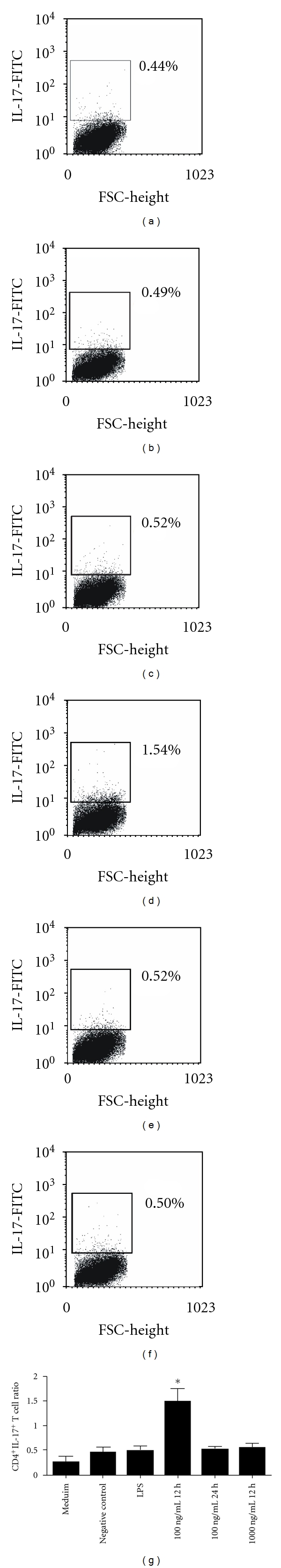
The number of IL-17-expressed CD4^+^T cell stimulated by HMGB 1. The conditions were designed as before. The cells were collected; before 6h, 1 *μ*L monosion, 5 *μ*L 10 ng/mL PMA, and 1 *μ*L 1 Mm/mL inon were added. (a) Presents medium control, stimulated for 12 h; (b) Presents independence protein, stimulated for 12 h; (c) Presents LPS, stimulated for 12 h; (d) Presents 100 ng/mL HMGB 1 stimulated for 12 h; (e) Presents 100 ng/mL HMGB 1 stimulated for 24 h; (f) Presents 1000 ng/mL HMGB 1 stimulated for 12 h.

Flow cytometry analysis showed that the ratio of IL-17-producing cells (Th17) was up to the maximum (1.50 ± 0.43%), while 100 ng/mL of HMGB1 was used for 12 h, it shown significant difference compared with other groups (*P*  <  0.05) (Figure 5).

## 4. Discussion

Th17 cells and their specific transcription factor or related cytokines are being recognized as important mediators in inflammatory and autoimmune diseases including RA, but relatively little is known about HMGB1 roles and the relationship between Th17 and HMGB1 in RA. In the present study, we found that in RA patients, the mRNAs of HMGB1, ROR*γ*t, and IL-17 in PBMCs and the levels of HMGB1, IL-17, and IL-23 in plasma were increased, and there was a positive correlation between HMGB1- and Th17-cell, especially in active phase of RA. Furthering analysis showed that HMGB1 and Th17 related factors also had the positive correlation with other RA clinical related detections. To study the relationship of HMGB1 and Th17 cell, we explored the function of HMGB1 on CD4^+^T cells *in vitro* and observed that rHMGB1 could enhance the ratio of Th17 cell. Neither the same source protein nor the endotoxin LPS had the function. We revealed that HMGB1-triggered the RA may via the Th17 pathway in RA pathogenesis.

There are two pathway of HMGB1 transit from intracellular to extracellular, one is secreted by activated innate immune cells, the other is released by the death or apoptosis cells [13–16]. HMGB1 in milieu was involved in the innate and adaptive immune system [7]. Previous data showed that HMGB1 could prime the naïve CD4^+^ T lymphocytes toward T helper 1 phenotype. Increasing evidence indicated that HMGB1 acts as an early inflammatory mediator in the pathogenesis of arthritis [11, 19]. 

Th17 cells and their effector cytokines are being recognized as important factors in organ-specific autoimmune diseases, especially which were thought mediating by Th1 cells before [20–25]. Th17 cells have emerged as critical effector cells in EAE pathogenesis [20, 21]. HMGB1 is a potent inducer of several proinflammatory cytokines, such as IL-1*β* and IL-6, which were considered as crucial mediators in inducing of Th17 cells [26]. Recently, Liu reported that HMGB1 can induce IL-23 through TLR4 pathway and IL-23 can enhance the IL-17 levels [27]. Philippa indicates that IL-23/IL-17 axis exist in the pathogenesis of RA [28]. HMGB1 also played important roles in other autoimmune diseases as well as in acute allograft rejection [29–32]. 

In the present study, we not only confirmed the previous results, but also indicated that the HMGB1 involved in pathogenesis of RA. There is a positive correlations between HMGB1 and Th17 or other clinical index. Our data from FACS also showed that HMGB1 might upregulate CD3^+^CD8^−^IL-17^+^T cells in RA patients, and also in our* in vitro *study, we observed that HMGB1 directly acted on CD4^+^ T cells to enhance IL-17 production following activation by CD3 and CD28 mAbs, which was consistent with our recent report [33]. In brief, our data provide a strong association between increased Th17 activity and HMGB1 in RA, and HMGB1 may upregulate Th17 cells *in vivo* or *in vitro*, which opens a new avenue in the studies of RA immunotherapy and pathogenesis.

## Figures and Tables

**Table 1:** The primers used in this study.

Gene	Sequence(5′–3′)	Length (bp)
HMGB1	5′-GATGGGCAAAGGAGATCCTA-3′ 5′-CTTGGTCTCCCCTTTGGGGG-3′	233
ROR*γ*t	5′-CCTGGGCTCCTCGCCTGACC-3′ 5′-TCTCTCTGCCCTCAGCCTTGCC-3′	171
IL-17	5′-CAAGACTGAACACCGACTAAG-3′ 5′-TCTCCAAAGGAAGCCTGA-3′	231
*β*-actin	5′-CACGAAACTACCTTCAACTCC-3′ 5′-CATACTCCTGCTTGCTGATC-3′	265

**Table 2:** The plasma concentration of HMGB1, IL-23, and IL-17 in RA patients.

	Active RA	Inactive RA	Health control
sample	48	32	50
HMGB1 (ng/mL)	8.420 ± 1.780*	6.315 ± 0.725	5.892 ± 0.901
IL-23 (pg/mL)	203.825 ± 99.321*	148.332 ± 91.278	103.825 ± 73.427
IL-17 (pg/mL)	409.239 ± 152.324*	188.325 ± 76.143	165.672 ± 46.238

*P  <  0.05 compared with healthy control.

**Table 3:** Correlations of HMGB1, IL-23, IL-17, and clinical index in the serum of active RA patients.

	HMGB1 (pg/mL)		IL-23 (pg/mL)		IL-17 (pg/mL)	
	*r*	*P*	*r *	*P*	*r*	*P*
Age	0.122	0.410	0.065	0.660	0.007	0.965
CRP (mg/dL)	0.894	0.000^#^	0.405	0.004^#^	0.454	0.001^#^
ESC (mm/h)	0.817	0.000^#^	0.328	0.02*	0.371	0.009^#^
RF (IU/mL)	0.707	0.000^#^	0.325	0.024*	0.370	0.010^#^

*P  <  0.05, ^#^ *P  *<  0.01 compared with healthy control.
